# A distinctive oral phenotype points to *FAM20A* mutations not identified by Sanger sequencing

**DOI:** 10.1002/mgg3.164

**Published:** 2015-10-04

**Authors:** James A. Poulter, Claire E. L. Smith, Gina Murrillo, Sandra Silva, Sally Feather, Marianella Howell, Laura Crinnion, David T. Bonthron, Ian M. Carr, Christopher M. Watson, Chris F. Inglehearn, Alan J. Mighell

**Affiliations:** ^1^ Section of Ophthalmology and NeuroscienceUniversity of LeedsLeedsUnited Kingdom; ^2^ School of DentistryUniversity of Costa RicaSan PedroCosta Rica; ^3^ BiologyMolecular CellularCentre (CBCM)University of Costa RicaSan PedroCosta Rica; ^4^ Paediatric NephrologyLeeds Teaching Hospitals NHS TrustLeedsUnited Kingdom; ^5^ Paediatric NephrologyNational Children's HospitalSan JoseCosta Rica; ^6^ Yorkshire Regional Genetics ServiceLeeds Teaching Hospitals NHS TrustLeedsUnited Kingdom; ^7^ Section of GeneticsSchool of MedicineUniversity of LeedsLeedsUnited Kingdom; ^8^ Department of Oral MedicineSchool of DentistryUniversity of LeedsLeedsUnited Kingdom

**Keywords:** Amelogenesis imperfecta, CNVseq, enamel renal syndrome, FAM20A

## Abstract

Biallelic *FAM20A* mutations cause two conditions where Amelogenesis Imperfecta (AI) is the presenting feature: Amelogenesis Imperfecta and Gingival Fibromatosis Syndrome; and Enamel Renal Syndrome. A distinctive oral phenotype is shared in both conditions. On Sanger sequencing of *FAM20A* in cases with that phenotype, we identified two probands with single, likely pathogenic heterozygous mutations. Given the recessive inheritance pattern seen in all previous *FAM20A* mutation‐positive families and the potential for renal disease, further screening was carried out to look for a second pathogenic allele. Reverse transcriptase‐PCR on cDNA was used to determine transcript levels. CNVseq was used to screen for genomic insertions and deletions. In one family, *FAM20A* cDNA screening revealed only a single mutated *FAM20A* allele with the wild‐type allele not transcribed. In the second family, CNV detection by whole genome sequencing (CNVseq) revealed a heterozygous 54.7 kb duplication encompassing exons 1 to 4 of *FAM20A*. This study confirms the link between biallelic *FAM20A* mutations and the characteristic oral phenotype. It highlights for the first time examples of *FAM20A* mutations missed by the most commonly used mutation screening techniques. This information informed renal assessment and ongoing clinical care.

## Introduction

Biallelic *FAM20A* mutations cause Amelogenesis Imperfecta (AI) and Gingival Fibromatosis Syndrome (AIGFS, OMIM: 614253) (O'Sullivan et al. 2011) and Enamel Renal Syndrome (ERS, OMIM: 204690) (Jaureguiberry et al. 2012; Wang et al. 2013). Both disorders involve hypoplastic AI in the primary and secondary dentitions, together with variable degrees of gingival hyperplasia, pulpal calcifications, and delayed tooth eruption (de la Dure‐Molla et al. 2014). In addition, nephrocalcinosis may subsequently develop with a variable age of onset that is incompletely defined. A total of 36 pathogenic *FAM20A* variants identified by Sanger sequencing have been published, including stop‐gain, frameshift, missense, and splice‐site mutations (Cho et al. 2012; Cabral et al. 2013; Kantaputra et al. 2014a,b; Wang et al. 2014; Cherkaoui Jaouad et al. 2015; Volodarsky et al. 2015). Some patients diagnosed with AIGFS have subsequently been recognized to have nephrocalcinosis. It has therefore been suggested that recessive *FAM20A* mutations are responsible for a single disease which should now be termed ERS (de la Dure‐Molla et al. 2014).

FAM20A is a member of a family of three homologous proteins, FAM20A, B, and C, known as the atypical secretory serine kinase family with sequence similarity 20. FAM20C is a Golgi casein kinase that phosphorylates key extracellular molecules involved in biomineralization (Tagliabracci et al. 2012), and mutations in the encoding gene (OMIM: 611061) cause recessively inherited Raine syndrome (OMIM: 259775) (Simpson et al. 2007). The oral phenotypes observed in individuals with either recessive mutations in *FAM20A* or *FAM20C* share features and may appear isolated in milder or earlier stages of disease (de la Dure‐Molla et al. 2014; Acevedo et al. 2015). Identification of the pathogenic mutation in these cases may therefore require sequencing of both genes. FAM20B, not as yet unequivocally associated with any inherited human condition, has been shown to be a Golgi kinase with a key role in phosphorylating xylose in glycosaminoglycan‐protein linkage region of proteoglycans. By comparison, little is known of FAM20A function. It is predicted to be a kinase with targets specific to mineralization, calcium transport, and proteoglycan synthesis (de la Dure‐Molla et al. 2014), but this remains to be confirmed experimentally. As such, FAM20A may have additional unknown functions. A recent study by Cui et al., however, suggests FAM20A lacks catalytic activity and therefore should be categorized as a pseudokinase (Cui et al. 2015). Instead they found FAM20A‐regulated phosphorylation of targets via activation of FAM20C. By forming a complex, kinase activity was enhanced leading to high levels of substrate phosphorylation. In cells lacking FAM20A, FAM20C kinase activity was inhibited. While further studies are required to understand this mechanism further, the identification of recessively inherited mutations in *FAM20A* as a cause of AIGFS and ERS suggests a key involvement of this protein in biomineralization.

In this study, we further investigated two families for whom only a single heterozygous missense mutation in *FAM20A* had been identified by Sanger sequencing of genomic DNA. The pathognomonic oral phenotype and autosomal recessive inheritance of *FAM20A* mutations strongly suggested the existence of a second deleterious allele. We therefore applied two further mutation detection screens; reverse transcriptase (RT)‐PCR to examine the *FAM20A* transcript and low‐coverage whole genome sequencing (CNVseq) which was used to identify dosage variants at the *FAM20A* locus. Using these approaches we were able to identify the second pathogenic alleles in both probands, which were undetected using standard Sanger sequencing. These findings had implications for ongoing clinical care.

## Materials and Methods

Affected individuals and relatives were recruited following informed consent in accordance with the principles outlined in the declaration of Helsinki, with local ethical approval. Genomic DNA samples were obtained using either Oragene^®^ DNA sample collections kits (DNA Genotek, Ontario, Canada) or via venous blood samples, using conventional techniques. Genomic DNA was extracted by salt (blood) or ethanol (saliva) precipitation.

Exons and flanking intronic sequences of *FAM20A* and *FAM20C* were amplified by the polymerase chain reaction (PCR) according to standard protocols. Primers were designed using ExonPrimer software (http://ihg.gsf.de/ihg/ExonPrimer.html) and are listed in Table S1.

Patient blood obtained for RNA transcript analysis was immediately stabilized with EDTA. RNA was extracted using the QIAamp RNA Blood Mini Kit system (Qiagen, Hilden, Germany). First‐strand cDNA synthesis was performed using the Moloney murine leukemia virus (M‐MLV) reverse transcriptase (Life Technologies, Carlsbad, CA) according to the suppliers' recommended instructions.

Copy number variant detection using low‐coverage whole genome sequencing data was performed as previously described (Wood et al. 2010; Watson et al. 2014). One microgram of genomic DNA was sheared using a Covaris S2 (Covaris, Inc., Woburn, MA) before Illumina compatible sequencing libraries were generated using NEBNext^®^ Ultra^™^ reagents (New England Biolabs, Ipswich, MA), following manufacturer's instructions. Twenty million single end 50‐bp reads were obtained for each sample using a HiSeq 2500 run in rapid mode. Raw sequence reads were aligned to an indexed human reference genome (hg19) using the Burrows–Wheeler aligner (bwa) v0.6.2 (Li and Durbin 2009) and duplicate reads were discarded using Picard v1.85 (http://picard.sourceforge.net). Coordinates of uniquely mapped test and reference reads were extracted from BAM files (Li et al. 2009) and counted into genomic windows containing equal numbers of reference reads. A read count adjustment was performed to compensate for the effect of local GC% variation, and the adjusted ratios of test to reference read counts were used as input to the R module DNAcopy v1.32.0 (Venkatraman and Olshen 2007), which segments the data into regions of equal copy number.

Whole exome sequencing was performed using 3 *μ*g of genomic DNA with the SureSelect All Exon v5 reagent according to the manufacturer's protocol. Sequencing was performed on an Illumina HiSeq 2500 across two lanes of a rapid mode flow cell using a 100 bp paired‐end protocol. Raw sequence reads for a pool of six exome libraries were demultiplexed and aligned to hg19 using bwa v0.6.2. Picard v1.85 removed duplicate reads. Dosage analysis was performed using FishingCNV (Shi and Majewski 2013) by comparing this sample to a cohort of 50 non‐AI‐affected controls.

## Results

### Presenting clinical features

**Figure 1:**
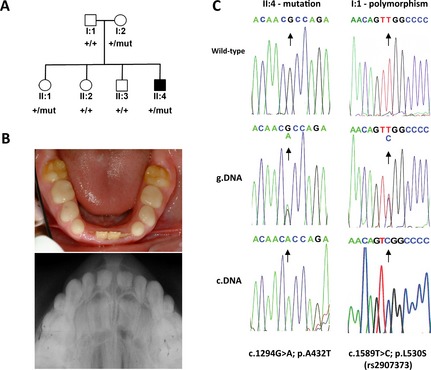
Dental phenotype and genetic analysis of Family 1. (A) Pedigree of Family 1 showing affected (shaded) and unaffected (unshaded) members of the family. The segregation of the wild‐type (+) and missense variant (c.1294G>A, NM_017565.3) p.A432T (mut) is shown below each family member. (B) Photograph (top) and dental X‐ray (bottom) showing the hypoplastic enamel phenotype of individual II:4. (C) Sequence electropherograms from the affected individual (II:4) showing the wild‐type allele (top), c.1294G>A mutation in genomic DNA (center) and in the cDNA (bottom). The mutation is heterozygous in gDNA but homozygous in the cDNA suggesting the wild‐type allele is not transcribed. Sequencing of the *FAM20A* polymorphism (c.1589T>C, NM_017565.3; p.L530S) in the unaffected father (I:1), who does not carry the c.1294A>G mutation, also showed it to be heterozygous in the genomic DNA (center) but homozygous in the cDNA (bottom), which is consistent with one the allele not being transcribed.

**Figure 2:**
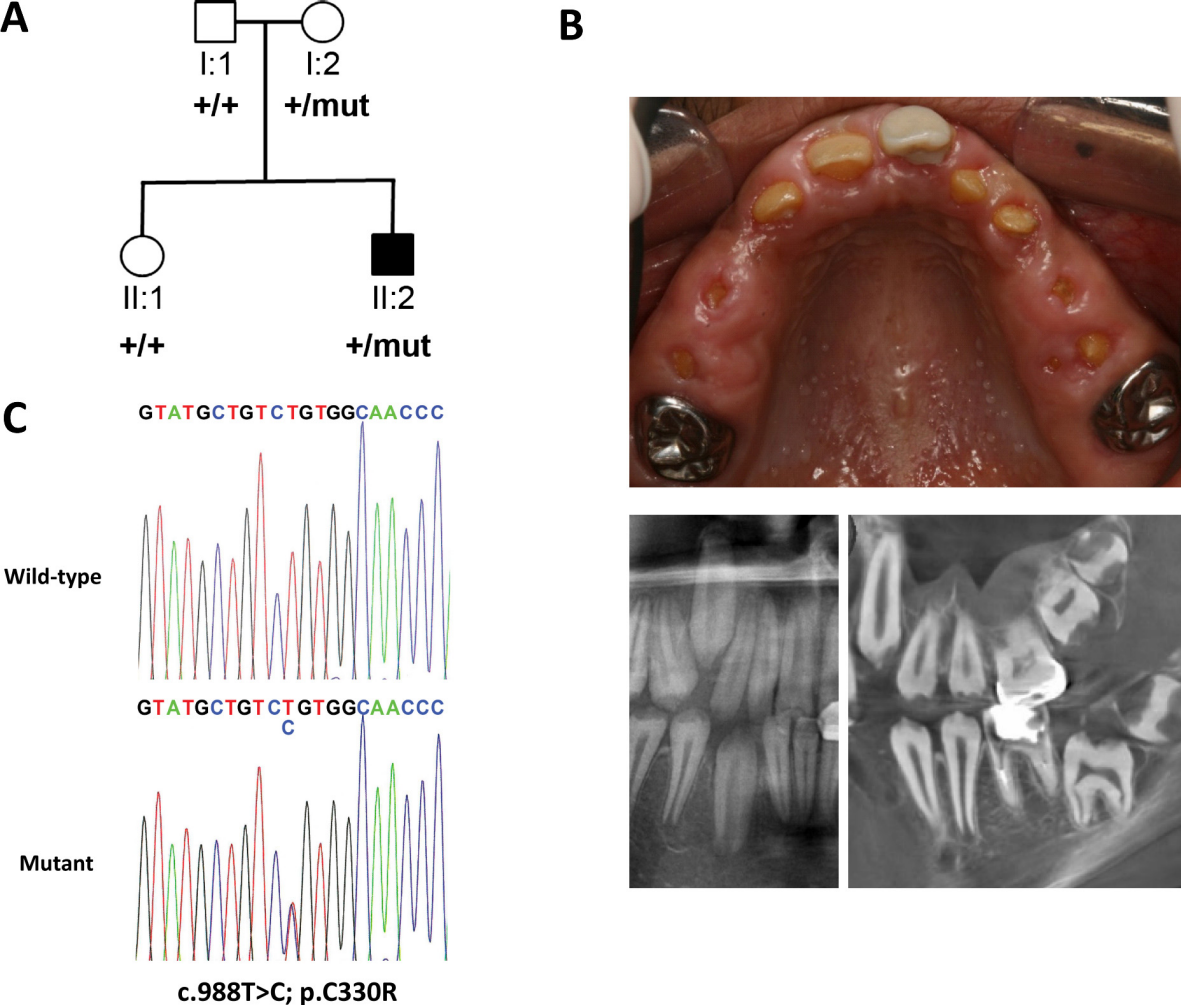
Dental phenotype and genetic analysis of Family 2. (A) Pedigree of Family 2 showing affected (shaded) and unaffected (unshaded) individuals. The segregation of the wild‐type (+) and missense variant (c.988T>C, NM_017565.3) p.C330R (mut) is shown below each family member. (B) Photograph (top) and dental imaging (bottom: left, panoramic; right, cone beam CT) showing the hypoplastic enamel phenotype and on the clinical image, mild gingival enlargement of individual II:2. (C) Electropherogram showing the wild‐type allele (top) and heterozygous c.988T>C mutation (bottom) identified in Family 2 (top).

The proband of Family 1 is a 17‐year‐old male born of unrelated parents of different ethnic origins (Pakistani and English) (Fig. 1A). He presented to clinic with oral features consistent with mutations in *FAM20A* (Fig. 1B). The proband of Family 2 is a 15‐year‐old male born of unrelated parents from Costa Rica (Fig. 2A). He presented to clinic with hypoplastic AI and mild gingival enlargement suggestive of mutations in *FAM20A* (Fig. 2B). In both families, no further symptoms were reported and no other family members had enamel abnormalities.

### Genomic analysis

All coding exons and flanking intronic sequences of *FAM20A* (NM_017565.3) were PCR amplified and sequenced directly by Sanger sequencing in both families.

In Family 1, this identified a novel heterozygous missense variant (c.1294G>A; p.A432T) in exon 9 (Fig. 1C). This variant is not present in dbSNP 138 and alters a residue that is fully conserved in orthologues and FAM20B (Fig. S1). The variant was predicted damaging by 4/5 bioinformatic prediction software packages tested (Table S2). Segregation analysis revealed that it was inherited from the maternal allele in the proband (Fig. 1A). An unaffected sibling (II:1) had also inherited the variant. No additional variants were identified in *FAM20A* nor its closest relative *FAM20C,* as would be expected for a recessive disease.

To determine whether a second mutation affecting splicing was present, leukocyte RNA from the affected individual and his father was obtained and first‐strand cDNA synthesis performed. RT‐PCR across *FAM20A* revealed no aberrant splicing. However, unexpectedly the c.1294G>A variant appeared homozygous in the cDNA of the proband (Fig. 1C). As the father is not a carrier of the c.1294G>A variant, a common SNP (c.1589T>C, p.L530S, rs2907373) in *FAM20A* exon 11 was genotyped instead. This was found to be heterozygous in the genomic DNA but homozygous in cDNA (Fig. 1C). These observations are consistent with only a single FAM20A allele being transcribed in the father, and the same phenomenon is evident in the affected son. In order to find an explanation for the failure in the transcription of an allele, we sequenced both 5ꞌ and 3ꞌ UTRs as well as conserved regions located 5ꞌ to *FAM20A*. No variation which could potentially cause a failure in transcription was identified. Despite further investigation by CNVseq of the affected individual no explanation could be identified for the lack of transcription.

In Family 2, screening of *FAM20A* identified a novel heterozygous missense variant (c.988T>C; p.C330R) in exon 7 (Fig. 2C) that is absent in dbSNP 138 and is fully conserved in orthologues and paralogues (Fig. S1). This was predicted to be damaging by all bioinformatic prediction software tested (Table S2). Segregation of this variant revealed that it was inherited from the mother. An unaffected sibling (II:1) was also heterozygous for it, but as with Family 1, no further variants were identified in *FAM20A,* or its paralogue *FAM20C*. Screening of leukocyte cDNA also revealed no observable sequence abnormalities, with the *FAM20A* variant being heterozygous in the cDNA of the proband as well as in the genomic DNA.

To determine whether the second mutation was a copy number variation, we performed low‐coverage whole genome sequencing (CNVseq) on genomic DNA of the affected individual. These data revealed increased dosage (1.5 × diploid) for a ~54.7 kb segment spanning the first four exons of *FAM20A* (approximately chr17:66,543,172‐66,597,963; Fig. S2). To verify this result, whole exome sequencing was performed on a second aliquot of DNA from the same individual. The resulting data were aligned to the human hg19 genome and dosage analysis was performed using FishingCNV by comparing this sample to a cohort of 50 non‐AI‐affected controls. Exomes from all controls and the AI‐affected sample had been captured, sequenced and analyzed using consistent methodologies to ensure that resulting files were comparable. This verified the presence of a duplication across *FAM20A* (spanning an approximate genomic location between chr17:66,548,013‐66,596,807) with a Holm adjusted *P*‐value of 1.27×10^−11^ (Table 1).

**Table 1:** List of regions with significant *P*‐values (<0.05) following analysis of WES data using FishingCNV

Location	Gene	Seg.mean	*P*‐value	Adjusted *P*‐value
chr11:22396300–22399286	*SLC17A6*	0.5547	9.50E‐23	2.16E‐19
chr17:66548013–66596807	*FAM20A*	0.5526	5.59E‐15	1.27E‐11
chr2:152235877–152267041	*TNFAIP6*	0.3731	2.18E‐08	4.95E‐05
chr2:196915910–196922840	*DNAH7*	0.5968	1.51E‐07	0.000343691
chr17:57663513–57676844	*DHX40*	0.6386	2.78E‐07	0.000630243
chr2:216226277–216226802	*FN1*	0.3568	9.74E‐07	0.002208401
chr2:223806214–223806372	*ACSL3*	0.6371	8.86E‐07	0.002009279
chr2:234178647–234178713	*ATG16L1*	0.7008	8.56E‐07	0.001942038
chr17:78360489–78362489	*RNF213*	−0.666	1.20E‐06	0.00272912
chr2:204131236–204154599	*CYP20A1*	0.3354	2.19E‐06	0.004967592
chr11:46702036–46703732	*ARHGAP1*	−0.3511	2.57E‐06	0.005813784
chr10:127422640–127426558	*C10orf137*	0.2957	2.87E‐06	0.006508269
chr13:33249980–33250096	*PDS5B*	0.7288	4.22E‐06	0.009545158
chr11:68350510–68350597	*PPP6R3*	0.5933	4.58E‐06	0.01036926
chr22:42293055–42294785	*SREBF2*	−0.6842	5.74E‐06	0.01297131
chr17:79768700–79769007	*GCGR*	−0.661	6.78E‐06	0.015316085
chr1:243736227–243736350	*AKT3*	0.6555	8.54E‐06	0.019287744
chr11:10820538–10820660	*EIF4G2*	0.625	8.97E‐06	0.020264384
chr3:197701912–197702982	*LMLN*	0.442	1.02E‐05	0.022952466
chr20:36561940–36561991	*VSTM2L*	0.625	1.11E‐05	0.025099307
chr5:102490373–102490647	*PPIP5K2*	−0.7072	1.67E‐05	0.037756914
chr7:31016895–31016937	*GHRHR*	0.6681	1.97E‐05	0.04446605

Regions are arranged in ascending order of adjusted *P*‐value. Only regions with significant Holm adjusted *P*‐values are shown. Seg.mean represents the mean log‐ratio of coverage between the 50 control exome BAM files and the affected exome BAM file.

### Renal evaluation

In light of molecular findings, both probands were assessed by pediatric nephrology specialists. The proband of Family 1 was assessed at the age of 15 and the proband of Family 2 was assessed at the age of 13. Investigations included urinalysis, blood biochemistry, and renal ultrasound. No abnormal findings were noted.

## Discussion

A distinctive clinical phenotype can be indicative of the causative mutated gene, however, Sanger sequencing and WES will not identify all disease‐causing mutations. In our cohort of eight families with orodental features consistent with biallelic mutations in *FAM20A*, six had biallelic *FAM20A* mutations (Jaureguiberry et al. 2012). Each of the remaining two families had single heterozygous missense *FAM20A* mutations. We hypothesized that within the spectrum of clinical features associated with biallelic *FAM20A* mutations, a second mutation should be present in each family that was not detectable by Sanger sequencing. This was important to investigate given the potential for renal involvement in the absence of all the orodental features that may be present.

Further analysis of the *FAM20A* locus revealed that the affected individual in Family 1 has inherited a paternal allele that does not express a *FAM20A* transcript, leaving only the maternal allele carrying a missense mutation present in mRNA from whole blood. In Family 2 a ~54.7 kb duplication encompassing exons 1–4 of *FAM20A* was identified as the second pathogenic mutation by CNVseq.

Regulating transcription in many differentiated cell types is an intricate task that requires a combination of multiple different transcriptional regulatory proteins. Cis‐acting regulatory sequences (e.g., the TATA box) are located adjacent to the genes they regulate, and are responsible for binding of RNA polymerase. Additionally, remote enhancers (RE's) exist which are located further away from the target gene but are equally vital to transcription. It is therefore possible that mutation of such an RE for *FAM20A* is present in Family 1, preventing transcription of *FAM20A*. Previous studies have found RE's as far as 20 kb away from the target gene meaning their identification can be difficult (Ghiasvand et al. 2011). In this study, we attempted to identify promoter or enhancer regions nearby to *FAM20A,* however, sequencing of these regions were negative or inconclusive. The reason for the unexpressed *FAM20A* allele in Family 1 therefore remains elusive.

Whole genome sequencing (WGS) is increasingly used for the analysis of DNA. Compared to WES, a wider variety of analyses can be performed, in part due to bias in PCR amplification of individual exons in WES, which leads to a heterogeneous profile of read coverage compared to WGS (Gilissen et al. 2014; Meynert et al. 2014). Low‐coverage whole genome sequencing is a reliable alternative to array comparative genomic hybridization for detecting large (>40 kb) structural variations that is comparable in cost (Hayes et al. 2013). In this study, CNVseq of an affected individual from Family 2 revealed a large duplication spanning *FAM20A*. Whole exome sequencing of the proband confirmed the presence of the putative duplication using an alternative wet‐laboratory technique and informatics algorithm. It is likely that the falling cost of DNA sequencing will enable WGS to be carried out at sufficient depth for it to be used as a first line investigation. This would allow a dosage analysis such as CNVseq to be undertaken concurrently with traditional point mutation or small insertion deletion variant detection (Meynert et al. 2014). Furthermore, the recent implementation of paired‐end 250‐bp HiSeq read lengths may make it possible to identify breakpoint‐spanning reads, thus allowing the characterization of duplications such as that reported for Family 2 to be mapped at nucleotide resolution.

Both missense variants identified in this study are in the highly conserved predicted kinase domain of FAM20A (based on homology to FAM20C) (Tagliabracci et al. 2012). Amino acid p.C330 is one of 12 cysteine residues in FAM20A, all of which are fully conserved in orthologues and paralogues. Cysteine residues form disulfide bonds which play a key role in forming the specific tertiary structure of the mature protein, so substitution of p.C330 is likely to affect the protein structure. Residue p.A432 lies within the Greek key domain at the C‐terminus of FAM20A, which is commonly found in *γ*‐crystallins of the human lens and plays a key role in binding calcium (Rajini et al. 2001). The substitution of a nonpolar alanine with a polar threonine is likely to interfere with this domain.

In light of the ERS clinical phenotype both families in this study underwent renal evaluation by nephrology specialists after confirmation of biallelic *FAM20A* mutations. No renal abnormalities were observed. This might imply a correlation between milder missense mutations and lack of nephropathology, but previous reported findings do not support this hypothesis. The absence of a clear relationship between *FAM20A* genotype and renal phenotype may reflect the influence of genetic modifiers or environmental factors such as diet. Given the progressive nature of the renal phenotype, a single renal assessment in childhood should not be interpreted as an absence of renal involvement. Ongoing monitoring is indicated until there is a better understanding of how to predict if and when renal nephrocalcinosis of clinical significance will develop.

In summary, we investigated two families with a distinctive orodental phenotype previously associated with recessive mutations in *FAM20A*, in whom only a single missense mutation had been identified by Sanger sequencing. Screening of *FAM20A* cDNA in family 1 revealed that the apparently wild‐type allele was not transcribed, whereas CNVseq of an affected individual from Family 2 revealed a large duplication encompassing exons 1–4 of *FAM20A*. These findings confirm the link between biallelic *FAM20A* mutations and the characteristic oral phenotype and give insights into the classes of mutations missed by the most commonly used mutation screening techniques, together with possible methodologies for identifying them. This insight informed renal assessment and ongoing clinical care.

## Conflict of Interest

The authors declare no conflict of interest.

## Supporting information


**Table S1.** Primers designed using ExonPrimer to amplify the exons and flanking intronic sequence of *FAM20A* (NM_017565.3) and *FAM20C* (NM_020223.3).
**Table S2.** Summary of bioinformatic analyses undertaken to predict the pathogenic nature of the missense mutations identified in FAM20A (NP_060035).

**Figure S1.** Conservation of the missense variants identified in this study.

**Figure S2.** Identification of a heterozygous duplication in *FAM20A* of Family 2.
