# Supplementary material for: A distinctive oral phenotype points to FAM20A mutations not identified by Sanger sequencing
Source: Mol Genet Genomic Med. 2015 Oct 4;3(6):543–9. doi: 10.1002/mgg3.164 (PMC4694127; doi:10.1002/mgg3.164)
Supplement: Supplementary file 1 — Table S1. Primers designed using ExonPrimer to amplify the exons and flanking intronic sequence of FAM20A (NM_017565.3) and FAM20C (NM_020223.3). Table S2. Summary of bioinformatic analyses undertaken to predict the pathogenic nature of the missense mutations identified in FAM20A (NP_060035). [file MGG3-3-543-s001.doc]

***Supplementary Tables***

**Supplementary Table 1** – **Primers designed using ExonPrimer to amplify the exons and flanking intronic sequence of *FAM20A* (NM_017565.3) and *FAM20C* (NM_020223.3).** Primers used to amplify cDNA (RT) were designed using Primer3.

| **FAM20A** | | **Forward Primer (5**′**-3**′**)** | | **Reverse Primer (5**′**-3**′**)** | | **Size (bp)** |  |
| --- | --- | --- | --- | --- | --- | --- | --- |
| Ex 1 | | TCCAGGCACCTCTTCTGC | | TCAGGAAACTCGAGACTGGG | | 574 |  |
| Ex 2 | | CTTGTGGAATAAATGAAAGGATG | | GGTGTCCATGTCAAGCCTCT | | 399 |  |
| Ex 3 | | TCCCCTCTGGTGATCTGAAC | | GAGTGGGTTTTGGGGTTTG | | 221 |  |
| Ex 4 | | TTGAAGGAACAGAGTCACAAGC | | TGGCAGAAAGACTTTAGGTCACT | | 248 |  |
| Ex 5 | | GTTTTCCTTTCTCGCTGGTC | | TGAGGGTCTGTCTAGCCACC | | 262 |  |
| Ex 6 | | CCAGCCAATTCCTAATGTGC | | GATGAATGGAGGGGTGGAC | | 285 |  |
| Ex 7 | | CCTAGGCCAGGAAGATGTCA | | GCCAAGCTAGCAACAAGTCC | | 332 |  |
| Ex 8 | | ATCAGGGAGGCAGGGATT | | AGGTCACGGGGAACCCTA | | 280 |  |
| Ex 9 | | TCCCTGGAGAGAACTCAGGT | | GTGGTGGAGGCCCTCATTT | | 267 |  |
| Ex 10 | | AGAGGAGAGAGGAAGCCTCA | | ACTGGAAGCCCTTCAGACCT | | 221 |  |
| Ex 11 | | TGTGGGGAAAGAAGTTCAGG | | CCAGCAGTAACAGGTGGGAG | | 435 |  |
|  |  | |  | |  | | |
| **FAM20C** | **Forward Primer (5**′**-3**′**)** | | **Reverse Primer (5**′**-3**′**)** | | **Size (bp)** | | |
| Ex 1-1 | GCACCGATGGACCTTGAC | | GGTCGTGGGGTCTTAGGG | | 507 | | |
| Ex 1-2 | GGCCCAACAAGCACACG | | GCCCCTGAACCTCTCTACAC | | 436 | | |
| Ex 2 | ATCTGCACTTGCTTGAACCC | | GGCCTCCCAGACCTCTCTAA | | 392 | | |
| Ex 3 | GACCATGCCCAGAGGACC | | AAAACACCCTGGGAGGAGAC | | 290 | | |
| Ex 4 | TGAGGAACCCAGCACGTC | | AGGACGGCCTCACTCACC | | 250 | | |
| Ex 5 | CTTATTTGGAGGCAGGGAC | | TCAGCACCCTGGTGTGGA | | 674 | | |
| Ex 6 | GGCCGTGAGACCACAGGT | | AGCGGTCATCTCACACAGG | | 399 | | |
| Ex 7-8 | TGCCGCAGTGTTTCTCTTCT | | CAGGTGGGCTGCAGGTAG | | 593 | | |
| Ex 9 | GTGTCGGGTACAGGCAGGT | | GCTGTGGCCTCCTCTGTCT | | 236 | | |
| Ex 10 | TCCCTCTCACTTTCTCTCGC | | TGTCCTATGAGACCTGGGGA | | 495 | | |
| RT | CTGGCCCAACAAGCACAC | | CCCGTAATTCTGGAAGGTCA | | 508* | | |

*Expected size in cDNA

**Supplementary Table 2** - **Summary of bioinformatic analyses undertaken to predict the pathogenic nature of the missense mutations identified in FAM20A (NP_060035).** URLs: PolyPhen2, <http://genetics.bwh.harvard.edu/pph2/> ; MutationTaster, <http://www.mutationtaster.org/> ; Blosum62; PROVEAN, <http://provean.jcvi.org/> ; MutPred, <http://mutpred.mutdb.org/> .

‎

| **Mutation** | **PolyPhen2** | **MutationTaster** | **Blosum62*** | **PROVEAN** | **MutPred** |
| --- | --- | --- | --- | --- | --- |
| p.C330R | 1.00  Probably Damaging | 0.99  Disease Causing | -3 | -11.385  Deleterious | Deleterious Probability  0.973 |
| p.A432T | 1.00  Probably Damaging | 0.99  Disease Causing | 0 | -2.473  Neutral | Deleterious Probability  0.790 |

*Blosum62 scores range from +3 to -3 with negative scores being more likely to be damaging substitutions.

**Supplementary References**

Adzhubei, I. A., S. Schmidt, L. Peshkin, V. E. Ramensky, A. Gerasimova, P. Bork, A. S. Kondrashov and S. R. Sunyaev (2010). "A method and server for predicting damaging missense mutations." Nature methods **7**(4): 248-249.

Choi, Y., G. E. Sims, S. Murphy, J. R. Miller and A. P. Chan (2012). "Predicting the functional effect of amino acid substitutions and indels." PloS one **7**(10): e46688.

Henikoff, S. and J. G. Henikoff (1993). "Performance evaluation of amino acid substitution matrices." Proteins **17**(1): 49-61.

Li, B., V. G. Krishnan, M. E. Mort, F. Xin, K. K. Kamati, D. N. Cooper, S. D. Mooney and P. Radivojac (2009). "Automated inference of molecular mechanisms of disease from amino acid substitutions." Bioinformatics **25**(21): 2744-2750.

Schwarz, J. M., C. Rodelsperger, M. Schuelke and D. Seelow (2010). "MutationTaster evaluates disease-causing potential of sequence alterations." Nature methods **7**(8): 575-576.
