# Supplementary figures and images for: A distinctive oral phenotype points to FAM20A mutations not identified by Sanger sequencing
Source: Mol Genet Genomic Med. 2015 Oct 4;3(6):543–9. doi: 10.1002/mgg3.164 (PMC4694127; doi:10.1002/mgg3.164)

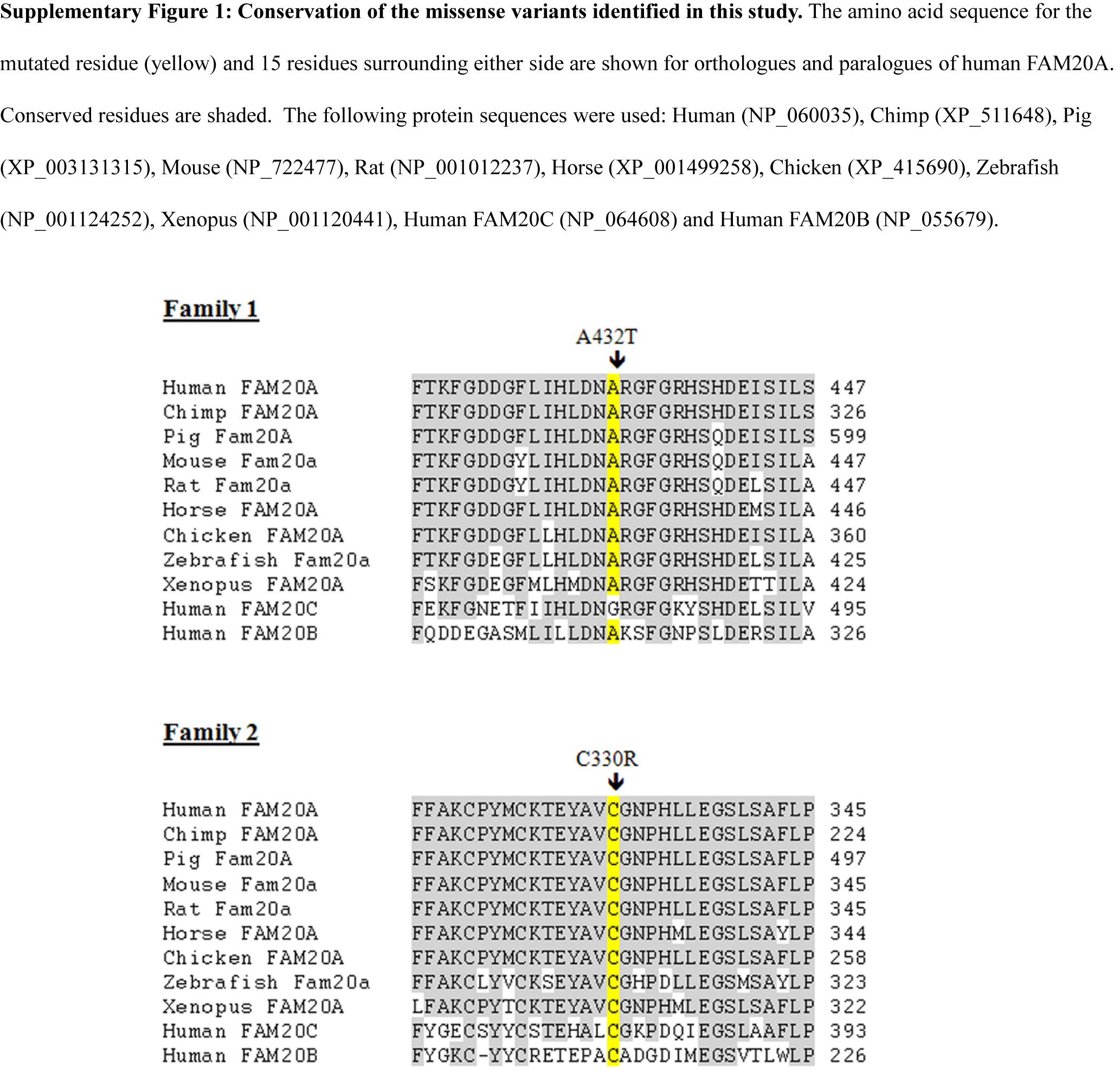

Supplement: Supplementary file 2 — Figure S1. Conservation of the missense variants identified in this study. [file MGG3-3-543-s002.tif]

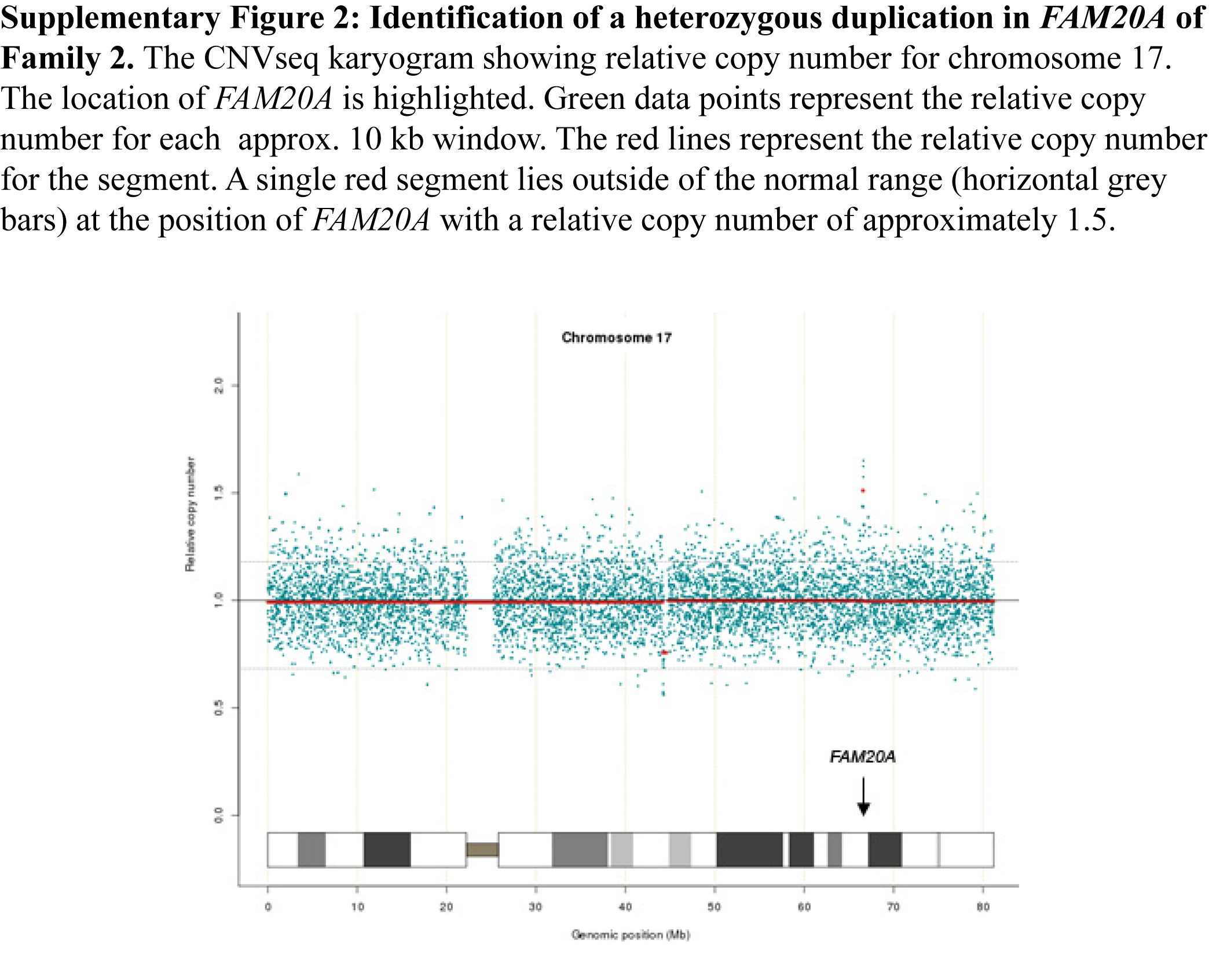

Supplement: Supplementary file 3 — Figure S2. Identification of a heterozygous duplication in FAM20A of Family 2. [file MGG3-3-543-s003.tif]
